# Bioactive Compounds Extracted from Saudi Dates Using Green Methods and Utilization of These Extracts in Functional Yogurt

**DOI:** 10.3390/foods12040847

**Published:** 2023-02-16

**Authors:** Kashif Ghafoor, Md. Zaidul Islam Sarker, Fahad Y. Al-Juhaimi, Isam A. Mohamed Ahmed, Elfadil E. Babiker, Mohammed S. Alkaltham, Abdullah K. Almubarak

**Affiliations:** 1 Department of Food Science and Nutrition, College of Food and Agricultural Sciences, King Saud University, Riyadh 11451, Saudi Arabia; 2 Food Science Program, Cooperative Research, Extension and Education Services, Northern Marianas College, Saipan, MP 96950, USA

**Keywords:** bioactive compounds, date flesh extract, green extraction methods, functional yogurt, yogurt quality

## Abstract

The bioactive compounds of four Saudi date flesh extracts (Ambara (AF), Majdool (MF), Sagai (SF), and Sukkari (SKF)) prepared using different extraction methods—namely, supercritical fluid extraction (SFE), subcritical CO_2_ extraction (SCE), and Soxhlet extraction (SXE)—were evaluated. A total of 19 bioactive compounds were detected in extracts prepared using SFE and SCE methods, whereas less than 12 compounds were detected in extracts obtained using the SXE method. Both the date variety and extraction method affected the phenolic profile of date flesh extract (*p* ≤ 0.05). The apparent viscosity, surface color, and bioactive properties of yogurt were affected by both date flesh extracts and storage duration in varied magnitudes (*p* ≤ 0.05). The incorporation of date flesh extracts into yogurt formulations increased the total phenolic content (TPC), DPPH antiradical activity, viscosity, and redness (a*) and decreased the lightness (L*) and yellowness (b*) of the developed product (*p* ≤ 0.05). The elongation of storage time progressively (*p* ≤ 0.05) reduced the pH, TPC, DPPH antiradical activity, bacterial counts, and L* and b* values and increased the acidity, syneresis, viscosity, and a* values with few exceptions. Date flesh extracts can improve the health quality of yogurt without major influence on the sensory attributes while stored at 4 °C.

## 1. Introduction

Bioactive phytochemicals are present in different parts of plants including fruits and vegetables. Phytochemicals are linked with certain health benefits such as against the development of chronic diseases. Hence, their role carries significant importance. These are non-nutritive biological compounds from fruits, vegetables and grains, and more than 5000 phytochemicals have been identified from various plant materials with the possibility of a huge number still being unexplored [1]. In order to study their functions and beneficial health effects, these compounds need to separated, purified and properly identified. Different in vitro and in vivo studies have been reported to study their health benefits. However, it had been reported that phytochemicals may have multiple mechanisms for their roles in addition to their generally reported antioxidant effects. Phytochemicals are generally categorized as phenolics, alkaloids, nitrogen-containing compounds, phytosterols and carotenoids among which phenolic compounds are the most studied ones in terms of their effects on human health [1,2,3]. The utilization of these phytochemicals depends also on the techniques used for their recovery from plant materials followed by chemical characterization, separation and use in functional foods and nutraceuticals, isolation, and application [4]. Such techniques can be categorized broadly into two groups: conventional methods such as Soxhlet, maceration and hydrodistillation and non-conventional or modern techniques that include the supercritical fluid (SFE), subcritical CO_2_ (SCE), ultrasound-assisted, enzyme-assisted, and microwave-assisted extraction, among others [4,5]. The modern methods have the advantage of using no or environmentally safe extraction solvents that include ethanol, water and CO_2_ [6,7]. Moreover, the modern methods may yield extracts containing more contents or the variety of bioactive compounds, and hence, such extracts may be utilized in food and nutraceutical products [4].

The date palm (*Phoenix dactylifera* L.) is an important fruiting plant in various countries around the globe. The date fruit is generally considered healthy and a good source of energy [8]. The occurrence of different bioactive phytochemicals has been reported in date fruit that may be the source for different health-promoting effects associated with the consumption of date fruit. In general, the quantification of date phytochemicals is generally carried out for total phenolic, total flavonoids, total anthocyanins and total carotenoids contents using spectrophotometric procedures [9,10,11]. There are also available reports on the quantification of pure compounds in date using advanced analytical techniques such as the high-performance liquid chromatography (HPLC) technique. The date fruit has been previously analyzed using an HPLC method [12] and different polyphenols either in their free (protocatechuic, vanillic, syringic, and ferulic acids) or bound (gallic acid, protocatechuic acid, p-hydroxybenzoic acid, vanillic acid, caffeic acid, syringic acid, p-coumaric acid, ferulic acid, and o-coumaric acid) state were reported. In another study, the HPLC technique was applied to detect polyphenolics in Khalas, Sukkari and Ajwa date varieties cultivated in Saudia Arabia, and caffeic acid (0.5–0.7 mg/100 g), catechin (0.5–0.75 mg/100 g) and rutin (0.4–0.8 mg/100 g) were detected [10]. Another study [13] reported the use of liquid chromatography-electrospray ionization tandem mass spectrometry (LC-ESI/MS/MS) procedures to identify polyphenols in Deglet noor dates. It was observed that thirteen flavonoid glycosides of luteolin, quercetin, and apigenin were present, and their certain structural attributes were further explained. However, no quantitative data were given. In a previous study [11], four different date varieties (Sukari, Ambara, Majdool and Sagai) were subjected to three different extraction techniques, namely SFE, SCE and Soxhlet (SX) on selected process conditions. The extracts obtained from each fruit type were analyzed for total phenolics, total flavonoids, total carotenoids and total anthocyanins and were found rich in phenolic and flavonoid compounds. The extracts also demonstrated appreciable antioxidant and free radical scavenging activities.

Due to changing consumer demand and the need for the development of new foods with additional health benefits (functional foods), the food industry is undergoing transitions and using new trends in product development. The development of functional food is aimed at not only fulfilling hunger but also providing additional health benefits. This trend is also correlated with increased life expectancy and the desire of the elderly for an improved life quality [14]. There is an increasing trend in the manufacture and consumption of yogurt in the 21st century. One of the reasons for this increase is the addition of fruit ingredients as a source of prebiotics, flavor, color and natural antioxidants in yogurt [14]. In a recent study [15], roasted barley powder (1.5–3.0%) was added to prepare a yogurt drink and increments in certain functional properties were observed. The technological properties, sensory and color attributes also scored higher than the control (without barley powder). The current research aims at the identification and quantification of bioactive compounds (phenolic acids and flavonoids) in extracts prepared using SFE, SCE and SXE techniques from the flesh of four different (Ambara, Majdool, Sagai and Sukari) date fruits using liquid chromatography-mass spectrometry (LCMS). Selected extracts were used in the development of a functional yogurt product, and different physicochemical properties in relation to the addition of extracts from date flesh were also investigated.

## 2. Materials and Methods

### 2.1. Raw Materials

Locally produced Sukari, Ambara, Majdool and Sagai date fruits were obtained from the Seasonal Date Fruit Market, Riyadh, Saudi Arabia. After separating from seeds manually, the date flesh was subjected to vacuum drying at 50 °C. The samples were then ground using a grinder (Panasonic, Shah Alam, Malaysia). Ingredients for yogurt preparation were obtained from the local market. For analytical work, different chemicals and standards were obtained from Sigma-Aldrich (St. Louis, MO, USA).

### 2.2. Extraction of Phytochemicals

The extracts from the flesh of four different date fruits were prepared using conventional (Soxhlet) and modern (supercritical CO_2_ or SFE and subcritical CO_2_ or SCE) methods as described previously [11]. Briefly, 25 g of date flesh powdered samples were first extracted using the Soxhlet (SX) method, in which n-hexane was used as a solvent and extraction was carried out for 10–12 h at 70 °C. To carry out extraction using SFE, the extraction conditions were set at 52.5 °C extraction temperature, 27.50 MPa extraction pressure and 5 mL/min CO_2_ flow rate. These SFE conditions were previously optimized [11]. The SCE process involved the use of 250 extraction cycles, a pressure of 6.8 MPa and a temperature of 29 °C. A 150 g powdered sample of date flesh was first soaked with 75 mL of cosolvent and then placed in SCE setup for extraction. Liquid CO_2_ (6 kg) and 95% ethanol were used as solvent and cosolvent in SCE process, respectively [11]. The extracts prepared from all the methods were dried and stored at −20 °C in airtight and light-proof containers until further analyses and use in functional foods.

### 2.3. Liquid Chromatography-Mass Spectrometry (LC-MS) 

An aliquot of ~15–70 mg of samples was used to estimate flavonoids and phenolic acids in extracts obtained using different Soxhlet, SFE and SCE methods. The analysis was conducted using a high-performance liquid chromatography system (Nexera X2, Shimadzu, Tokyo, Japan). The system was connected to a mass spectrometer (QTRAP 6500, Sciex, MA, USA). The phytochemicals from dates were separated using a reverse-phase C18 column (100 mm × 3.0 mm; 3.5 μm particle size) (Eclipse XDB, Agilent Technologies, Santa Clara, CA, USA). Acetic acid (2%) in water (A) and 100% acetonitrile (B) were used as a mobile phase in this study. The gradient conditions were set as reported previously [16]. A 1 µL sample was injected into the system, while the column temperature was kept ambient. An Ion Drive Turbo V ion source (ESI) was used for positive and negative ion modes in the MS/MS system (Sciex, Framingham, MA, USA). The ESI source temperature was set at 450 °C; the voltage of ion spray was 5500 and −4500 V, and the pressure of ion source gas 1, ion source gas 2 and curtain gas was 50, 50 and 25 psi, respectively. The collision gas was medium. A multiple reaction monitoring (MRM) mode was used to operate the MS/MS system with the optimized collision. A Sciex Analyst 1.6.3 software (Sciex, Framingham, MA, USA) package was used for the optimization of analytical parameters (MRM transition molecular and product ions; the energies for ionization and collision; declustering, entrance and collision cell exit potentials) and data processing [16]. The normalization of the data was accomplished based on internal standard GR24 spiked in the sample before separation to account for experimental variation. The identification of bioactive compounds was carried out through the comparison of results with those of analytical standards. 

### 2.4. Preparation of Functional Yogurt

Drinking yogurt was prepared in 7 different batches including control. The treatments included 6 yogurt samples each containing 1 g of dried extract from Majdool (MF) and Sukari (SKF) date flesh obtained using 3 different extraction techniques (SFE, SCE and SX). These two varieties were selected due to their high phenolic contents and better antioxidant properties [11]. A control sample was also prepared to which no extract was added. The date extracts were mixed with 14 g of skim milk powder followed by the addition of distilled water (100 mL). After 5 min of homogenization, samples were placed in a water bath for 30 min to pasteurize at 85 °C followed by cooling to 44 °C. Afterwards, bacterial starter culture (2.5% *v*/*v*) consisting of *Streptococcus thermophilus* and *Lactobacillus delbrueckii* subsp. *bulgaricus* (Hansen, Hoersholm, Denmark) was added in a 1:1 ratio. The samples were then incubated at 44 °C for 4 h until they were fully coagulated (pH 4.6). The curd so formed was first cooled down to 4 °C in a refrigerator and then homogenized to form the liquefied drinkable yogurt. The final yogurt samples were then stored at 4 °C for carrying out various quality studies.

### 2.5. Physical Analysis of Yogurt 

The total solids or °Brix, pH, syneresis and acidity were measured [17]. The analysis of apparent viscosity was carried out using a viscometer (Brookfield Eng Labs. Inc., Stoughton, MA, USA) and results were presented as Pa.s (pascal-second). The color of the yogurt samples was determined using a colorimeter (colorimeter (Minolta Camera Co., Osaka, Japan). The colorimeter calibration was carried out using a standard white plate. The color parameters evaluated were L* representing lightness or darkness, a* showing redness or greenness and b* demonstrating the yellowness and blueness of the samples. All physical parameters were evaluated in triplicates.

### 2.6. Sensory Evaluation of Yogurt

The sensory evaluation was carried out to determine the consistency, taste, odor and appearance of the yogurt on a 9-point scale where 1 was disliked extremely and 9 was liked extremely. The sensory panel included 20 semi-trained evaluators, and their informed consent was obtained about the use of natural date extracts in the samples. Samples were coded before presenting them to the evaluators, and water was used during the evaluation of different treatments.

### 2.7. Microbiological Evaluation of Yogurt

The microbial analysis of yogurt samples was carried out to estimate the population of lactic acid bacteria (LAB) at different storage intervals. A yogurt sample of 1 g was added to sterile peptone water (9 mL) containing 0.1 g of peptone in 100 mL water. The samples were then serially diluted using peptone water (1:10). Afterwards, 1 mL of each of the diluted samples was added to M17 agar (6.5 pH) for estimation of *S. thermophilus*) and MRS agar (pH 5.6) for the estimation of the population of *S. thermophilus* and *L. bulgaricus*, respectively. The yeast/mold count was carried out using potato dextrose agar. The inoculated plates were incubated at 45 °C under aerobic and anaerobic conditions for *S. thermophilus* and *L. bulgaricus*, respectively. The colony counting for LAB was performed after 3-day incubation. The yeast/mold count was performed after 5-day incubation plated at 37 °C. The results of microbial enumeration were presented as log CFU/g yogurt. The counting of yeast, and molds in all yogurt types was also carried out (no yeast and mold detected in freshly prepared yogurt) to confirm the process hygiene.

### 2.8. Determination of Total Phenolics and Antioxidant Activities in Yogurt

The total phenolic compounds (TPC) of yogurt samples were evaluated using the previously described method [18] with slight modifications. A 5 g yogurt sample was mixed with 40 mL of 50% methanol and agitated for 1 h at room temperature. After filtration, the mixture was centrifuged for 10 min at 5000× *g*. The supernatant was separated and analyzed for TPC using Folin–Ciocalteu reagent (FCR). Followed by the incubation of a mixture containing supernatant, FCR and sodium carbonate (20%) at room temperature for 2 h, the absorbance values were recorded 765 nm using a spectrophotometer (PerkinElmer, Waltham, MA, USA). Gallic acid was used as standard and results were expressed as mg of gallic acid equivalents (GAE).

The antioxidant activity was measured as DPPH (1,1 diphenyl 2 picrylhydrazyl) radical scavenging activity (%) as described previously [19] with some modifications. A sample of 1 mL supernatants from the TPC assay was mixed with 2 mL of DPPH solution (1 mg/100 mL methanol). After room temperature incubation for 10 min, DPPH radical scavenging of the sample was measured using absorbance values (AV) of the sample and control (blank) measured at 517 nm.

(1) $$Radical\;scavenging\;\left(\%\right)=\frac{AV_{Control}-AV_{Sample}}{AV_{Control}}\times 100$$

### 2.9. Statistical Analysis

All the extraction, analytical and product development studies were carried out in triplicates, and data were represented by means ± standard deviation (SD). A Duncan multiple range test was also used to compare mean values, and data were considered significantly different when *p* < 0.05.

## 3. Results and Discussion

### 3.1. Bioactive Compounds of Date Flesh Extracts Prepared Using Different Extraction Methods

The results of phenolic acids and flavonoid compounds in date flesh extracts of four date varieties (Ambara flesh (AF), Majdool flesh (MF), Sagai flesh (SF) and Sukari flesh (SKF)) as influenced by supercritical fluid (SFE), subcritical CO_2_ (SCE), and Soxhlet (SXE) extraction methods are shown in Table 1. The LC/MS chromatograms of all the extracts are presented in Figure S1. Generally, date flesh is considered as a source of bioactive compounds, and the composition of phenolic and flavonoid compounds is varied among date varieties and extraction methods [11]. A total of 19 bioactive compounds were identified in extracts of AF, MF, and SF prepared using SFE and SCE methods, whereas only 15 and 16 compounds were detected in SKF by both extraction methods. In addition, a few compounds were extracted from all date flesh samples with the SXE method. These findings indicate that both the date variety and extraction method affect the phenolic profile of date flesh extracts. Moreover, different amounts of bioactive compounds were identified in date flesh extracts, and the amount of each compound is greatly influenced by the date variety and extraction method. Factors such as date variety, growing and postharvest conditions, ripening stage, environmental conditions, and extraction and determination methods of phenolic compounds could likely affect the phenolic compounds in date flesh extracts [11]. Despite the lower number of extracted phenolic compounds, the SXE method extracted the highest amounts of vanillic, syringic, ferulic, and cinnamic acids from all date types compared to other extraction methods. Among these compounds, the highest (*p* ≤ 0.05) amounts of vanillic, ferulic, and cinnamic acids were observed in MF, whereas that of syringic acid was found in SKF extracted with the SXE method. In the SXE method, extraction was conducted using *n*-hexane at 70 °C for 10–12 h, and under these extreme conditions, thermal stable phenolic compounds could be extracted in high amounts due to the thermal disruption of a cell wall matrix, thereby enhancing the release of more soluble phenolic compounds. In addition, the elongated exposure of cellular fluid to repeated solvent recycling increased the liquefying properties of soluble components and hence increased the release and extractability of phenolic compounds [20,21]. The limited number of phenolic compounds in SXE is likely due to the degradation of sensitive compounds during long exposure to high temperature. SKF extracted with SCE possessed the highest (*p* ≤ 0.05) levels of rutin, quercetin-3-glucoside, luteolin, quercetin, apigenin, kaempferol, gallic, protocatechuic, chlorogenic, caffeic, and p-coumaric acids. The higher quantities of these phenolic compounds in SKF suggest that the application of the SCE method might have had positive effects on the recovery of these compounds. Moreover, the highest (*p* ≤ 0.05) levels of catechin, epicatechin, and naringenin were observed in SF extracted with the SCE method. Overall, SCE is the suitable extraction method for most of the phenolic compounds from date flesh followed by SFE, whereas the SXE extracted less amounts of a wide range (19 compounds) of phenolic compounds from all date types. The extraction of polar and nonpolar phenolic compounds by the combination of temperature and pressure used in the SCE and SFE methods could be the reason for the increased extraction of more phenolic compounds than SXE [11,22]. Previous reports have shown that date fruit is considered as a rich source of phenolic compounds, and a wide range of phenolic compounds including those reported in our study was commonly determined in date fruit extracts [23]. In this study, SKF exhibited the richest source of phenolic compounds followed by MF; therefore, the date flesh of these two date varieties was selected for the development of functional yogurt. These types of dates are the most popular date varieties and are known as a nutritionally rich source of phenolic compounds [23,24,25].

### 3.2. Effect of Date Flesh Extracts Prepared Using Different Extraction Methods on the Physicochemical Properties of Functional Yogurt

The results of physicochemical properties of functional yogurt containing date extracts of Majdool (MF) and Sukkari (SKF) fleshes prepared using different extraction (SFE, SCE, and SXE) methods and stored at 4 °C for 20 days are shown in Table 2. Generally, the incorporation of date flesh extracts (SFEMF, SFESKF, SCEMF, SCESKF, SXEMF, and SXESKF) into yogurt formulation affected the physicochemical properties in different magnitudes during cold storage. The total soluble solids (TSS) of yogurt were not influenced by date flesh extracts and storage duration with the exception of those formulated with SFEMF and SXESKF, which showed a slight (*p* ≤ 0.05) reduction in TSS at the end of storage that could be due to the metabolic process of fermenting microorganisms by which some nutrients could be degraded and hence resulted in the reduction in TSS [26].

The pH and acidity of yogurt were not affected by the incorporation of date flesh extracts in the yogurt formulations, whereas they were influenced by storage duration (*p* ≤ 0.05) (Table 2). A similar trend of pH changes was found in yogurts fortified with natural extracts from date palm spikelets [27] and red ginseng [28]. The elongation of storage time progressively (*p* ≤ 0.05) reduced the pH and increased the acidity of control and date flesh extracts-containing yogurts except for the acidity of SFEMF and SFESKF yogurts, which was not affected by storage duration. The insignificant changes in the acidity of SFEMF and SFESKF yogurt samples during storage could be due to the presence of some bioactive compounds in these extracts that control the metabolic activity of lactic acid bacteria and maintain the steady bacterial growth during storage as reported previously in yogurt fortified with date spikelets extract [27] and jujube pulp [29]. The reduction in pH and increase in the acidity of control and date flesh-fortified yogurt samples during storage is likely due to the metabolic activity of lactic acid bacteria that hydrolyze lactose and fiber into lactic and uronic acids, respectively, thereby increasing the acidity and reducing the pH of stored yogurt samples [27,30]. In agreement with our findings, previous reports have shown similar trends in pH and acidity during the storage of yogurt samples fortified with different natural antioxidant extracts [27,31,32]. The acidity values reported in this study ranged between 0.75% and 0.93% lactic acid, which is within the minimum Codex Alimentarius recommended standard range (0.60–1.5% lactic acid) for the acidity of fermented dairy products [33].

Syneresis is one of the important physical attributes of yogurt that can affect the storage stability and consumer acceptability of the product, and fewer changes in syneresis is preferable during the storage of yogurt [34]. The syneresis of fresh yogurt was not affected by the incorporation of date flesh extracts; however, stored yogurt showed varied (*p* ≤ 0.05) influences of date flesh extracts on the syneresis (Table 2). Increasing the storage time concurrently increased (*p* ≤ 0.05) the syneresis of all yogurt samples to the maximum values at day 20 of storage with the highest overall syneresis value being observed in control yogurt, whereas SFEMF, SFESKE, and SCESKF yogurts showed low syneresis at the end of the storage period, suggesting that these date flesh extracts reduced the syneresis changes during the cold storage of functional yogurts and hence maintain the physical quality attributes of the fortified yogurts. The increase in acidity during the storage of yogurt could reduce the net negative charges of casein micelles and its colloid stability and thereby increase the separation of whey protein and water from yogurt clots. This ultimately results in an increment in yogurt syneresis [35]. A similar increase in the syneresis during storage has been reported in yogurt fortified with date palm spikelets [27], and olive leaf hot water [35] extracts and fortified yogurts showed fewer changes in syneresis during storage, which has also been reported in different studies [27,32,36].

The apparent viscosity of yogurt was significantly (*p* ≤ 0.05) influenced by both date flesh extracts and storage duration (Table 2). The incorporation of date flesh extract into yogurt formulations increased the viscosity of the product, except for the SCESKF yogurt sample, which showed similar viscosity with that of fresh control yogurt; however, it showed different viscosity levels of stored yogurts. The increase in the viscosity of fortified yogurt is probably due to the interaction of yogurt proteins with phenolic compounds of date flesh extract, thereby forming a firm three-dimensional network with increased gel viscosity [32]. Similarly, previous reports indicated that the incorporation of moringa leaf [31], olive leaf [35], and chia seed [37] extracts into yogurt formulation significantly increased the viscosity of the fortified yogurts compared to the control. During storage, the viscosity of all yogurt samples showed a progressive increase, reaching the highest values at the end of storage (*p* ≤ 0.05). The highest viscosity was observed in the control yogurt followed by SEFSKF and SXESKF yogurts at the end of storage, suggesting that the extraction method also affected the viscosity of the developed yogurts. Factors such as the post-acidification and formation of exopolysaccharide (EPS) by LAB cells under storage conditions, the interaction of formed EPS will milk components, and the interaction of phenolic compounds of date flesh extracts with yogurt protein could modify the yogurt gel network structure and thus increase the viscosity of the fortified yogurt during storage [38,39]. In agreement with our findings, a similar increasing trend of the viscosity of plain and fortified yogurts during cold storage has been reported [27,38,40,41].

The total phenolic content (TPC) and DPPH antiradical activity of yogurt were influenced positively by date flesh extract addition and negatively by storage time elongation (Table 2). The incorporation of date flesh extracts into yogurt formulations significantly (*p* ≤ 0.05) increased the TPC and DPPH antiradical activity of the functional products. The highest TPC values were observed in SFEMF yogurt followed by SCEMF yogurt samples, whereas the lowest values were found in control yogurt throughout the entire storage period (*p* ≤ 0.05). The highest DPPH antiradical activity value was seen in fresh SCEMF yogurt samples followed by that of fresh SFEMF and SCESKF yogurts (*p* ≤ 0.05). Among yogurts containing different date flesh extracts, SXESKF yogurts showed the lowest TPC followed by SFESKF and SXEMF, suggesting that the extraction method and date type also influenced the TPC of functional yogurts. The increase in the TPC and DPPH antiradical activity of yogurt fortified with date flesh extracts is due to high level of phenolic compounds in date flesh extracts prepared using different extraction methods [11]. These findings demonstrated that the incorporation of date flesh extracts into yogurt formulations could improve the bioactive properties and antioxidant potentials and thus improve the nutritional and health quality attributes of fortified functional yogurt. Similarly, improvements of the TPC and antioxidant activity of yogurt have been reported following the fortification of yogurt samples with *Pleurotus ostreatus* aqueous extract [42], moringa leaf extract [31], chia seed extract [37], olive leaf extract [35] and date palm spikelets extract [41]. During storage, the TPC and DPPH antiradical activity of all yogurts showed a concurrent reduction with the increasing storage time, reaching minimum values at the end of storage (*p* ≤ 0.05). The reduction in TPC and the DPPH antiradical activity of yogurt samples during storage could be due to the influence of oxygen and metabolic enzymes from lactic acid bacteria and the degradation of certain polyphenols. Additionally, the reduced extractability of phenolic compounds from a yogurt matrix may also be due to the formation of complexes between phenolic compounds of date flesh extracts and yogurt proteins (protein–polyphenols interactions) [32,42,43,44]. In agreement with our findings, previous reports showed a concurrent reduction in TPC and DPPH antioxidant activity as the storage time progressed of yogurts fortified with different natural antioxidant extracts [35,42,43,44].

Starter culture lactic acid bacteria (*Lactobacillus bulgaricus* and *Streptococcus thermophiles*) counts in fresh and stored yogurt samples were high (7.35–9.71 log CFU/mL) and were not affected by the addition of date flesh extracts (Table 2). During storage, the counts of *L. bulgaricus* and *S. thermophiles* were progressively (*p* ≤ 0.05) reduced as the storage period elongated of control and SXESKF yogurts, whereas that of all other yogurts fortified with date flesh extracts remained constant during the first 10 days of storage and then reduced at the end of storage except for the count of *L. bulgaricus* in SFESKF, which increased at the end of storage. The decline of LAB counts during storage is likely due to the pH reduction and acidity increase in yogurt during storage [43], and similar observations on the reduction in LAB count during the storage of functional yogurts have been reported by various investigators [32,45]. The viable counts of yogurts fortified with date flesh extracts were maintained at ≥7.9 log CFU/mL, which were higher than the minimum standard counts (7 log CFU/mL) specified by Codex Alimentarius for probiotic yogurts [33], indicating the health potentials of developed functional yogurts. Generally, the incorporation of date flesh extracts into yogurt formulation improved the stability of lactic acid bacteria during the storage of functional yogurts and thereby improve the probiotic potentials of date extracts-containing yogurts.

### 3.3. Effect of Date Flesh Extracts Prepared Using Different Extraction Methods on the Surface Color Attributes of Functional Yogurt

Surface color is one of the physical quality attributes that influences the marketing and consumer preferences for yogurt [46]. Determining the surface color attributes of yogurts is of great importance from producers’ and consumers’ standpoints. The surface color attributes (lightness to darkness (L*); redness to greenness (a*) and yellowness to blueness (b*)) of functional yogurt fortified with extracts of Majdool and Sukkari date flesh prepared using different extraction methods (SFE, SCE, and SXE) and stored at 4 °C for up to 20 days are presented in Table 3. The L* values of yogurt were greatly reduced by the incorporation of date flesh extract and storage duration (*p* ≤ 0.05). The maximum L* values were seen in fresh and stored control yogurt, and the minimum L* value was observed in SXESKF yogurt at day 20 of storage. The redness (a*) values were increased (*p* ≤ 0.05) following the addition of date flesh extracts to yogurt formulations, and low (*p* ≤ 0.05) values were seen in control yogurts compared to fortified ones. In addition, the a* values of all yogurt samples were concurrently (*p* ≤ 0.05) increased as the storage period progressed, reaching maximum values at the end of the storage period. Among all yogurt samples, the highest a* value was observed in SCESKF yogurt followed by that of SCEMF at day 20 of storage, whereas the lowest values were seen in control yogurt on day 1 of storage (*p* ≤ 0.05). The yellowness (b*) values of yogurt samples were affected by the date flesh extracts and storage duration in a different manner. The highest b* values of all yogurt samples were seen in fresh yogurt samples, and as the storage time progressed, the b* values reduced to the lowest values at day 20 of storage (*p* ≤ 0.05). The reduction in L* values and increase in a* values in fortified yogurts is likely due to the brown color of the added date flesh extracts. Similarly, a reduction in L* values and an increase in a* values of yogurts fortified with natural antioxidant extracts have been reported in various studies [27,37,42]. The reduction in L* and b* values of yogurt samples during storage is probably due to the structural changes of the gel network structure, which reduced the free water on the surface of fortified yogurt and thereby reduced the lightness and yellowness values of the functional yogurts [27]. Whereas the increase in a* of fortified yogurt during storage could be attributed to the acidity development and structural modifications of yogurt’s three-dimensional structure that promote the release of red pigments of date flesh extracts from the yogurt matrix into the surface and thereby increased redness values during the storage of fortified yogurts [47]. In agreement with our results, previous reports showed a similar trend of surface color changes during the storage of functional yogurts [32,42,47].

### 3.4. Effect of Date Flesh Extracts Prepared Using Different Extraction Methods on the Sensory Attributes of Functional Yogurt

The sensory attributes (appearance, color, aroma, taste, texture, and overall acceptability) of functional yogurt fortified with extracts of Majdool and Sukkari date flesh prepared using different extraction methods (SFE, SCE, and SXE) and stored at 4 °C for up to 20 days are presented in Table 4. Generally, the incorporation of date flesh extracts into yogurt formulations did not affect the appearance, color, aroma, taste, texture, and overall acceptability of the fortified yogurt. Similarly, previous reports demonstrated that the incorporation of red ginseng extract [28] and date palm spikelets extract [41] into yogurt formulations did not affect the sensory attributes of the product. During storage, the sensory attributes were influenced by the date flesh extract and storage duration in different manners despite the general reduction in sensory attributes at the end of the storage period of all yogurt samples. The scores of the appearance, color, aroma, and texture of all yogurt samples were higher than 5 (the cutoff score) on the 9-point hedonic scale, indicating the high panelist preference for these attributes. The taste and overall acceptance scores of all yogurt samples were higher than the cutoff score with exception of the taste scores of SCESKF and SXESKF yogurt samples and the overall acceptance of SXEMF and SXESKF yogurt samples at the end of the storage period. These findings indicate that the incorporation of Soxhlet-extracted Sukkari date flesh extract into yogurt formulation could affect the overall acceptability of the stored yogurt, whereas the effect on fresh yogurt is negligible. The sensory scores for all sensory attributes of all yogurt samples are within the range of 4–9, which is recommended as an acceptable range for yogurt samples [48]. Overall, the findings of this study revealed that date flesh extracts improved the nutritional and health quality of yogurt without a major influence on the sensory attributes of the product during elongated storage at 4 °C.

## 4. Conclusions

This study concludes that date flesh is rich in phenolic compounds that were effectively recovered by using innovative green extraction (supercritical fluid extraction (SFE) and subcritical CO_2_ extraction (SCE)) as well as conventional Soxhlet extraction (SXE) methods. The quantity and composition of phenolic compounds in date flesh extracts are influenced by both date variety and extraction methods. The incorporation of date flesh extracts into yogurt formulations significantly improved the bioactive properties (TPC and DPPH antiradical activity), physical properties (viscosity and syneresis), and probiotic potential (LAB counts of more than 7.8 CFU/mL) and overcome adverse effects of prolonged storage on the overall quality attributes of the developed functional yogurt. In addition, the sensory acceptability of the developed functional yogurt was greatly conserved during storage as fewer changes in sensory attributes were observed in functional yogurts during storage. Overall, date flesh extracts can improve the nutritional and health quality of yogurt without a major influence on the sensory attributes of the product during refrigerated storage.

## Figures and Tables

**Table 1:** Phenolic acids and flavonoids (mg/100 g extract) as detected by LC/MS in flesh extracts of four date fruit varieties (Ambara flesh (AF), Majdool flesh (MF), Sagai flesh (SF) and Sukari flesh (SKF)) using supercritical fluid (SFE), subcritical CO_2_ (SCE) and Soxhlet (SXE) extraction methods.

Sample Name/Compounds	SFEAF	SFEMF	SFESF	SFESKF	SCEAF	SCEMF	SCESF	SCESKF	SXEAF	SXEMF	SXESF	SXESKF
Gallic acid	0.027 ± 0.01 d	0.023 ± 0.01 e	0.021 ± 0.01 e	0.033 ± 0.01 c	0.058 ± 0.02 b	0.055 ± 0.02 b	0.058 ± 0.01 b	0.170 ± 0.00 a	ND	ND	ND	ND
Protocatechuic acid	1.254 ± 0.38 c	2.467 ± 0.52 c	1.925 ± 0.65 c	0.478 ± 0.26 e	2.070 ± 0.72 c	4.711 ± 1.04 b	4.388 ± 1.18 b	13.439 ± 3.65 a	0.920 ± 0.08 d	0.531 ± 0.05 e	0.475 ± 0.35 e	0.783 ± 0.26 d
Catechin	0.011 ± 0.00 b	0.022 ± 0.00 b	0.038 ± 0.01 b	ND	0.019 ± 0.01 b	0.039 ± 0.01 b	0.081 ± 0.02 a	ND	ND	ND	ND	ND
Chlorogenic acid	0.005 ± 0.00 c	0.010 ± 0.00 b	0.010 ± 0.00 b	0.001 ± 0.00 c	0.013 ± 0.00 b	0.026 ± 0.01 b	0.025 ± 0.01 b	0.064 ± 0.01 a	ND	0.028 ± 0.01 b	ND	0.040 ± 0.01 b
Procyanidin B2	0.003 ± 0.00 e	0.006 ± 0.00 d	0.019 ± 0.00 b	ND	0.009 ± 0.00 c	0.012 ± 0.00 b	0.045 ± 0.01 a	ND	ND	ND	ND	ND
Vanillic acid	0.271 ± 0.05 j	0.387 ± 0.05 i	0.469 ± 0.15 hi	0.499 ± 0.16 hi	0.608 ± 0.05 h	0.949 ± 0.38 g	1.762 ± 0.45 f	2.581 ± 0.62 e	48.50 ± 5.09 b	58.69 ± 6.86 a	17.074 ± 5.46 d	38.608 ± 4.56 c
Caffeic acid	0.203 ± 0.06 d	0.258 ± 0.07 d	0.094 ± 0.03 b	0.047 ± 0.02 c	0.320 ± 0.14 c	0.442 ± 0.21 c	0.185 ± 0.08 d	1.655 ± 0.72 a	0.257 ± 0.06 d	0.211 ± 0.03 d	0.045 ± 0.05 c	0.395 ± 0.15 c
Syringic acid	0.073 ± 0.01 h	0.098 ± 0.03 h	0.123 ± 0.06 g	0.184 ± 0.06 g	0.167 ± 0.05 g	0.250 ± 0.09 g	0.490 ± 0.24 f	1.193 ± 0.51 e	10.25 ± 2.65 c	21.720 ± 4.65 b	3.512 ± 0.96 d	31.455 ± 6.71 a
Epicatechin	0.007 ± 0.00 c	0.009 ± 0.00 c	0.019 ± 0.00 b	ND	0.011 ± 0.01 b	0.015 ± 0.00 b	0.033 ± 0.01 a	ND	ND	ND	ND	ND
p-Coumaric acid	0.650 ± 0.05 c	1.128 ± 0.35 b	0.661 ± 0.26 c	0.183 ± 0.05 d	1.030 ± 0.25 b	2.088 ± 0.72 b	1.766 ± 0.58 b	4.181 ± 1.31 a	1.971 ± 0.29 b	1.072 ± 0.42 b	0.789 ± 0.52 c	0.873 ± 0.21 c
Ferulic acid	2.694 ± 0.14 i	6.020 ± 0.71 g	2.615 ± 0.72 i	1.406 ± 0.31 j	4.428 ± 1.31 h	11.043 ± 3.24 f	6.665 ± 1.95 g	22.778 ± 5.67 d	70.927 ± 10.34 b	106.932 ± 15.39 a	13.862 ± 2.65 e	72.421 ± 8.67 b
Rutin	0.638 ± 0.06 d	0.691 ± 0.13 d	0.809 ± 0.21 c	1.077 ± 0.51 bc	1.333 ± 0.56 b	1.472 ± 0.76 b	1.932 ± 0.35 b	4.725 ± 1.26 a	0.008 ± 0.00	0.019 ± 0.00 e	ND	0.095 ± 0.01 e
Quercetin-3-glucoside	0.455 ± 0.5 f	0.915 ± 0.08 e	0.793 ± 0.18 e	1.280 ± 0.49 d	0.847 ± 0.62	2.045 ± 0.85 b	1.878 ± 0.73 c	7.782 ± 2.36 a	0.031 ± 0.01 h	0.009 ± 0.00 i	ND	0.084 ± 0.01 g
Luteolin	0.014 ± 0.00 e	0.068 ± 0.03 c	0.028 ± 0.01 d	0.110 ± 0.09 b	0.041 ± 0.01	0.154 ± 0.04 b	0.056 ± 0.03	0.512 ± 0.18 a	0.002 ± 0.00 f	0.012 ± 0.00 e	ND	0.005 ± 0.00 g
Quercetin	0.007 ± 0.00 f	0.035 ± 0.01 d	0.025 ± 0.01 c	0.018 ± 0.01 e	0.023 ± 0.01 c	0.081 ± 0.03 b	0.045 ± 0.01 c	0.233 ± 0.05 a	ND	ND	ND	ND
Cinnamic acid	0.071 ± 0.02 g	0.080 ± 0.05 g	0.152 ± 0.06 f	0.088 ± 0.03 g	0.223 ± 0.06 e	0.283 ± 0.12 e	0.964 ± 0.35 d	0.341 ± 0.09 e	9.510 ± 2.68 b	15.502 ± 3.54 a	3.034 ± 1.05 c	3.389 ± 0.79 c
Naringenin	0.002 ± 0.00 e	0.003 ± 0.00 e	0.015 ± 0.00 c	ND	0.008 ± 0.00 d	0.014 ± 0.00 c	0.083 ± 0.05 a	0.021 ± 0.01 b	ND	ND	ND	ND
Apigenin	0.003 ± 0.00 d	0.005 ± 0.00 c	0.005 ± 0.00 c	0.011 ± 0.00 b	0.010 ± 0.00 b	0.015 ± 0.00 b	0.017 ± 0.00 b	0.029 ± 0.01 a	ND	ND	ND	ND
Kaempferol	0.014 ± 0.00 d	0.027 ± 0.01 c	0.009 ± 0.00 e	0.031 ± 0.01 c	0.040 ± 0.01 b	0.080 ± 0.02 a	0.028 ± 0.01 c	0.092 ± 0.02 a	ND	ND	ND	ND

Values are means ± SD of triplicate samples. Means not sharing a common letter(s) in a row are significantly different at *p* ≤ 0.05 as assessed by Duncan’s multiple range test. ND = not detected.

**Table 2:** Physicochemical parameters of functional liquid yogurt containing extracts from Majdool (MF) and Sukari flesh (SKF) obtained using supercritical fluid (SFE), subcritical CO_2_ (SCE) and Soxhlet (SXE) extraction techniques during storage at 4 °C for 20 days.

Parameter/Yogurt Sample	Storage Period (days)	Control	SFEMF Yogurt	SFESKF Yogurt	SCEMF Yogurt	SCESKF Yogurt	SXEMF Yogurt	SXESKF Yogurt
TSS (%)	1	12.62 ± 0.13 ^ap^	12.71 ± 0.19 ^ap^	12.58 ± 0.13 ^ap^	12.48 ± 0.16 ^ap^	12.48 ± 0.08 ^ap^	12.75 ± 0.11 ^ap^	12.68 ± 0.16 ^ap^
	10	12.55 ± 0.24 ^ap^	12.63 ± 0.21 ^ap^	12.63 ± 0.21 ^ap^	12.61 ± 0.16 ^ap^	12.45 ± 0.12 ^ap^	12.65 ± 0.13 ^ap^	12.55 ± 0.15 ^ap^
	20	12.42 ± 0.15 ^ap^	11.95 ± 0.13 ^bp^	12.36 ± 0.15 ^ap^	12.24 ± 0.15 ^ap^	12.08 ± 0.14 ^ap^	12.31 ± 0.10 ^ap^	11.96 ± 0.11 ^bp^
pH	1	4.98 ± 0.11 ^ap^	4.82 ± 0.10 ^ap^	4.78 ± 0.06 ^ap^	4.89 ± 0.09 ^ap^	4.79 ± 0.08 ^ap^	4.92 ± 0.05 ^ap^	4.89 ± 0.06 ^ap^
	10	4.31 ± 0.08 ^bp^	4.53 ± 0.09 ^bp^	4.66 ± 0.08 ^ap^	4.80 ± 0.18 ^ap^	4.68 ± 0.05 ^ap^	4.62 ± 0.06 ^ap^	4.28 ± 0.06 ^abp^
	20	4.01 ± 0.07 ^bp^	4.25 ± 0.03 ^cp^	4.18 ± 0.06 ^bp^	4.35 ± 0.10 ^bp^	4.17 ± 0.03 ^bp^	4.22 ± 0.10 ^bp^	4.09 ± 0.03 ^bp^
Acidity (%)	1	0.76 ± 0.06 ^bp^	0.75 ± 0.08 ^ap^	0.81 ± 0.03 ^ap^	0.79 ± 0.06 ^bp^	0.78 ± 0.08 ^bp^	0.76 ± 0.03 ^bp^	0.77 ± 0.05 ^bp^
	10	0.82 ± 0.06 ^ap^	0.81 ± 0.06 ^ap^	0.85 ± 0.05 ^ap^	0.85 ± 0.06 ^abp^	0.86 ± 0.09 ^abp^	0.88 ± 0.06 ^ap^	0.81 ± 0.02 ^abp^
	20	0.77 ± 0.05 ^bq^	0.83 ± 0.06 ^apq^	0.88 ± 0.08 ^apq^	0.92 ± 0.10 ^ap^	0.93 ± 0.06 ^ap^	0.89 ± 0.03 ^ap^	0.90 ± 0.04 ^ap^
Syneresis (%)	1	17.19 ± 0.85 ^cp^	17.06 ± 1.23 ^bp^	17.21 ± 0.82 ^cp^	17.12 ± 0.75 ^bp^	17.19 ± 0.57 ^cp^	17.23 ± 0.89 ^cp^	17.12 ± 0.72 ^bp^
	10	18.19 ± 0.48 ^bp^	17.23 ± 0.98 ^bq^	17.64 ± 0.65 ^bq^	17.66 ± 0.69 ^bq^	17.48 ± 0.49 ^bcq^	18.60 ± 0.72 ^bp^	17.79 ± 1.31 ^bp^
	20	20.10 ± 1.19 ^ap^	17.65 ± 0.36 ^as^	17.97 ± 1.14 ^as^	18.42 ± 0.63 ^ar^	17.73 ± 0.82 ^as^	19.72 ± 0.65 ^aq^	18.47 ± 1.12 ^ar^
Apparent viscosity (Pa.s)	1	148.95 ± 2.78 ^cs^	150.26 ± 3.36 ^ar^	152.36 ± 1.63 ^cp^	150.45 ± 1.36 ^cr^	148.69 ± 2.47 ^cs^	151.36 ± 2.92 ^cq^	151.66 ± 2.47 ^cq^
	10	152.35 ± 3.52 ^bs^	149.54 ± 4.25 ^bt^	155.23 ± 1.72 ^bq^	154.62 ± 2.64 ^br^	155.32 ± 3.16 ^bq^	156.82 ± 1.85 ^bp^	156.32 ± 2.39 ^bp^
	20	165.62 ± 1.67 ^ap^	150.63 ± 2.87 ^at^	162.45 ± 2.48 ^aq^	158.16 ± 1.88 ^ar^	157.59 ± 3.47 ^as^	158.76 ± 1.67 ^ar^	162.71 ± 3.17 ^aq^
Total Phenolics (mgGAE/100g)	1	3.98 ± 0.53 ^av^	28.56 ± 2.47 ^ap^	20.95 ± 1.66 ^at^	27.25 ± 2.11 ^aq^	25.36 ± 2.74 ^ar^	22.63 ± 1.37 ^as^	17.61 ± 1.63 ^au^
	10	3.65 ± 0.36 ^av^	26.52 ± 1.92 ^bp^	18.65 ± 1.28 ^bt^	25.64 ± 3.41 ^bq^	24.63 ± 1.83 ^br^	21.64 ± 1.54 ^bs^	15.54 ± 1.42 ^bu^
	20	3.02 ± 0.71 ^bt^	25.45 ± 3.42 ^cp^	17.62 ± 2.64 ^cr^	25.42 ± 2.58 ^bp^	19.35 ± 1.39 ^cq^	19.35 ± 1.62 ^cq^	15.48 ± 2.58 ^bs^
Radical scavenging (%)	1	11.56 ± 2.75 ^bu^	42.65 ± 5.27 ^aq^	35.89 ± 3.78 ^as^	48.79 ± 4.32 ^ap^	42.17 ± 2.75 ^aq^	37.56 ± 2.24 ^ar^	30.47 ± 2.72 ^at^
	10	12.48 ± 3.47 ^au^	40.65 ± 4.51 ^bq^	33.45 ± 4.62 ^bs^	45.36 ± 3.67 ^bp^	40.29 ± 3.72 ^bq^	34.62 ± 3.42 ^br^	27.92 ± 2.65 ^bt^
	20	11.61 ± 1.96 ^bv^	38.79 ± 4.88 ^cr^	30.78 ± 3.73 ^cs^	39.47 ± 1.11 ^cq^	40.62 ± 2.62 ^bp^	23.72 ± 1.76 ^cu^	25.18 ± 3.79 ^ct^
*Lactobacillus bulgaricus* (log CFU/mL)	1	8.85 ± 0.24 ^ap^	8.79 ± 0.25 ^ap^	8.69 ± 0.34 ^bp^	8.93 ± 0.07 ^ap^	8.84 ± 0.11 ^ap^	8.78 ± 0.34 ^ap^	8.86 ± 0.20 ^ap^
	10	8.07 ± 0.16 ^bp^	8.76 ± 0.17 ^ap^	8.52 ± 0.31 ^bp^	8.65 ± 0.18 ^ap^	8.73 ± 0.24 ^ap^	8.67 ± 0.31 ^ap^	8.17 ± 0.32 ^bp^
	20	7.35 ± 0.23 ^cr^	8.36 ± 0.22 ^bp^	8.91 ± 0.09 ^ap^	8.75 ± 0.22 ^ap^	8.62 ± 0.36 ^ap^	8.37 ± 0.52 ^ap^	7.89 ± 0.29 ^cq^
*Streptococcus thermophiles* (log CFU/mL)	1	9.62 ± 0.54 ^ap^	9.54 ± 0.39 ^ap^	9.42 ± 0.28 ^ap^	9.62 ± 0.37 ^ap^	9.67 ± 0.27 ^ap^	9.53 ± 0.36 ^ap^	9.71 ± 0.21 ^ap^
	10	8.36 ± 0.52 ^bp^	8.75 ± 0.24 ^ap^	8.87 ± 0.39 ^bp^	8.87 ± 0.41 ^bp^	8.29 ± 0.18 ^bp^	8.35 ± 0.15 ^bp^	8.52 ± 0.17 ^bp^
	20	7.85 ± 0.29 ^cp^	8.24 ± 0.63 ^bp^	8.65 ± 0.47 ^bp^	8.24 ± 0.24 ^bp^	8.21 ± 0.36 ^bp^	8.18 ± 0.24 ^bp^	8.09 ± 0.21 ^cp^

Values presented as a mean of triplicate samples (±SD). Means not sharing a common superscript(s) a, b, and c in a column or p, q, r, s, t, u and v in a row for each parameter are significantly different at *p* ≤ 0.05 as assessed by Duncan’s multiple range test. TSS = Total soluble solids.

**Table 3:** Color attributes of functional liquid yogurt containing extracts from Majdool (MF) and Sukari (SKF) obtained using supercritical fluid (SFE), subcritical CO_2_ (SCE) and Soxhlet (SXE) extraction techniques during storage at 4 °C for 20 days.

Color/Yogurt Sample	Storage Period (days)	Control	SFEMF Yogurt	SFESKF Yogurt	SCEMF Yogurt	SCESKF Yogurt	SXEMF Yogurt	SXESKF Yogurt
L*	1	86.52 ± 0.78 ^ap^	78.64 ± 0.47 ^ar^	77.98 ± 0.72 ^ar^	78.72 ± 0.75 ^ar^	79.24 ± 0.57 ^ar^	80.25 ± 0.34 ^aq^	80.47 ± 0.63 ^aq^
	10	83.45 ± 0.68 ^bp^	76.82 ± 0.75 ^bs^	75.32 ± 0.63 ^ct^	76.42 ± 0.25 ^bs^	78.54 ± 0.65 ^bq^	78.97 ± 0.95 ^bq^	77.65 ± 0.56 ^br^
	20	79.64 ± 0.72 ^cp^	75.96 ± 0.38 ^cs^	76.24 ± 0.22 ^br^	76.22 ± 0.36 ^br^	76.54 ± 0.57 ^cr^	77.36 ± 0.27 ^cq^	74.25 ± 0.28 ^ct^
a*	1	−2.25 ± 0.11 ^br^	−1.56 ± 0.04 ^bp^	−1.45 ± 0.08 ^ap^	−1.28 ± 0.04 ^bp^	−1.07 ± 0.02 ^cp^	−1.47 ± 0.03 ^ap^	−1.92 ± 0.08 ^aq^
	10	−2.09 ± 0.07 ^bs^	−1.24 ± 0.06 ^bq^	−1.22 ± 0.11 ^aq^	−1.16 ± 0.07 ^bq^	−0.87 ± 0.07 ^bp^	−1.24 ± 0.05 ^aq^	−1.75 ± 0.02 ^ar^
	20	−1.52 ± 0.06 ^as^	−0.89 ± 0.03 ^aq^	−1.08 ± 0.04 ^ar^	−0.85 ± 0.06 ^aq^	−0.52 ± 0.06 ^ap^	−1.05 ± 0.06 ^ar^	−1.63 ± 0.04 ^as^
b*	1	15.62 ± 0.14 ^ap^	14.25 ± 0.35 ^aq^	14.36 ± 0.22 ^aq^	14.62 ± 0.45 ^aq^	14.36 ± 0.47 ^aq^	14.58 ± 0.24 ^aq^	14.36 ± 0.34 ^aq^
	10	13.55 ± 0.31 ^bq^	13.98 ± 0.27 ^bp^	13.68 ± 0.36 ^bq^	13.25 ± 0.33 ^bq^	14.08 ± 0.31 ^ap^	13.98 ± 0.41 ^bq^	14.12 ± 0.24 ^ap^
	20	12.14 ± 0.15 ^cq^	13.74 ± 0.32 ^bp^	13.47 ± 0.18 ^bp^	13.42 ± 0.41 ^bp^	13.98 ± 0.41 ^ap^	13.69 ± 0.17 ^bp^	13.08 ± 0.19 ^bp^

Values presented as a mean of triplicate samples (±SD). Means not sharing a common letter(s) a, b, and c in a column or p, q, r, s and t in a row for each parameter are significantly different at *p* ≤ 0.05 as assessed by Duncan’s multiple range test. L* represents lightness; a* represents redness to greenness and b* represents yellowness to blueness.

**Table 4:** Sensory attributes of functional liquid yogurt containing extracts from Majdool (MF) and Sukari (SKF) obtained using supercritical fluid (SFE), subcritical CO_2_ (SCE) and Soxhlet (SXE) extraction techniques during storage at 4 °C for 20 days.

Attribute/Yogurt Sample	Storage Period (Days)	Control	SFEMF Yogurt	SFESKF Yogurt	SCEMF Yogurt	SCESKF Yogurt	SXEMF Yogurt	SXESKF Yogurt
Appearance	1	7.25 ± 0.62 ^ap^	7.34 ± 0.42 ^ap^	7.19 ± 0.46 ^ap^	7.35 ± 0.19 ^ap^	7.14 ± 0.64 ^ap^	7.15 ± 0.75 ^ap^	7.24 ± 0.84 ^ap^
	10	7.34 ± 0.38 ^ap^	7.02 ± 0.37 ^ap^	6.74 ± 0.52 ^ap^	6.98 ± 0.37 ^ap^	7.11 ± 0.41 ^ap^	6.68 ± 0.63 ^bp^	6.82 ± 0.37 ^ap^
	20	6.07 ± 0.57 ^bp^	5.98 ± 0.19 ^bp^	6.03 ± 0.39 ^bp^	6.15 ± 0.12 ^bp^	5.98 ± 0.36 ^bp^	6.22 ± 0.27 ^cp^	5.94 ± 0.43 ^bp^
Color	1	7.62 ± 0.56 ^ap^	7.35 ± 0.27 ^ap^	7.19 ± 0.42 ^ap^	7.55 ± 0.62 ^ap^	7.62 ± 0.42 ^ap^	7.51 ± 0.62 ^ap^	7.36 ± 0.69 ^ap^
	10	7.56 ± 0.64 ^ap^	7.28 ± 0.34 ^ap^	7.24 ± 0.36 ^ap^	7.08 ± 0.31 ^ap^	7.24 ± 0.38 ^ap^	7.12 ± 0.17 ^ap^	6.87 ± 0.51 ^bp^
	20	7.01 ± 0.37 ^bp^	6.08 ± 0.29 ^bq^	6.72 ± 0.61 ^bp^	6.58 ± 0.22 ^bp^	6.82 ± 0.17 ^bp^	6.54 ± 0.60 ^bp^	6.28 ± 0.33 ^cq^
Aroma	1	7.32 ± 0.23 ^ap^	7.48 ± 0.48 ^ap^	7.36 ± 0.39 ^ap^	7.68 ± 0.34 ^ap^	7.33 ± 0.11 ^ap^	7.66 ± 0.51 ^ap^	7.46 ± 0.74 ^ap^
	10	7.45 ± 0.11 ^ap^	7.11 ± 0.38 ^ap^	7.25 ± 0.43 ^ap^	6.96 ± 0.75 ^bp^	6.92 ± 0.63 ^bp^	6.78 ± 0.42 ^bp^	7.08 ± 0.67 ^ap^
	20	7.05 ± 0.53 ^ap^	6.89 ± 0.27 ^bp^	6.36 ± 0.61 ^bq^	6.37 ± 0.32 ^cq^	6.49 ± 0.34 ^cq^	6.17 ± 0.69 ^cq^	6.36 ± 0.61 ^bq^
Taste	1	6.88 ± 0.43 ^ap^	6.83 ± 0.42 ^ap^	6.78 ± 0.27 ^ap^	7.15 ± 0.42 ^ap^	6.85 ± 0.44 ^ap^	6.92 ± 0.24 ^ap^	6.59 ± 0.52 ^ap^
	10	6.56 ± 0.55 ^ap^	6.25 ± 0.65 ^ap^	6.66 ± 0.39 ^ap^	6.55 ± 0.15 ^bp^	5.76 ± 0.54 ^bq^	6.31 ± 0.82 ^ap^	5.66 ± 0.87 ^bq^
	20	5.25 ± 0.34 ^bp^	5.18 ± 0.31 ^bp^	5.24 ± 0.17 ^bp^	5.36 ± 0.39 ^cp^	4.99 ± 0.36 ^cp^	5.17 ± 0.24 ^bp^	4.87 ± 0.22 ^cp^
Texture	1	7.15 ± 0.61 ^ap^	7.26 ± 0.81 ^ap^	6.98 ± 0.39 ^ap^	7.36 ± 0.26 ^ap^	7.13 ± 0.47 ^ap^	6.69 ± 0.66 ^ap^	6.72 ± 0.68 ^ap^
	10	6.72 ± 0.37 ^bp^	6.74 ± 0.31 ^bp^	6.78 ± 0.24 ^ap^	6.59 ± 0.36 ^bp^	6.71 ± 0.97 ^bp^	6.39 ± 0.34 ^ap^	6.13 ± 0.41 ^bp^
	20	5.85 ± 0.25 ^cp^	5.32 ± 0.28 ^cp^	5.68 ± 0.62 ^bp^	6.13 ± 0.08 ^cp^	6.07 ± 0.42 ^cp^	5.36 ± 0.17 ^bp^	5.18 ± 0.39 ^cp^
Overall acceptability	1	7.35 ± 0.61 ^ap^	7.18 ± 0.37 ^ap^	7.29 ± 0.24 ^ap^	7.58 ± 0.40 ^ap^	7.19 ± 0.35 ^ap^	6.78 ± 0.61 ^ap^	7.04 ± 0.72 ^ap^
	10	6.58 ± 0.17 ^bp^	6.49 ± 0.62 ^bp^	6.29 ± 0.19 ^bp^	6.73 ± 0.37 ^bp^	6.25 ± 0.18 ^bp^	5.12 ± 0.38 ^bq^	5.29 ± 0.36 ^bq^
	20	5.56 ± 0.31 ^cp^	5.31 ± 0.27 ^cp^	5.68 ± 0.37 ^cp^	5.67 ± 0.11 ^cp^	5.45 ± 0.22 ^cp^	4.83 ± 0.27 ^cq^	4.62 ± 0.41 ^cq^

Values presented as a mean of triplicate samples (±SD). Means not sharing a common letter(s) a, b, and c in a column or p and q in a row for each parameter are significantly different at *p* ≤ 0.05 as assessed by Duncan’s multiple range test.

## Data Availability

No new data were created or analyzed in this study. Data sharing is not applicable to this article. Data is contained within the article or supplementary material.

## Supplementary Materials

The following supporting information can be downloaded at: https://www.mdpi.com/article/10.3390/foods12040847/s1, Figure S1: LC/MS chromatogram for phenolic acids and flavonoids in extracts from date flesh of four date fruit varieties (Ambara flesh (AF), Majdool flesh (MF), Sagai flesh (SF) and Sukari flesh (SKF)) using supercritical fluid (SFE), subcritical CO_2_ (SCE) and Soxhlet (SXE) extraction methods
