# Supplementary material for: Bioactive Compounds Extracted from Saudi Dates Using Green Methods and Utilization of These Extracts in Functional Yogurt
Source: Foods. 2023 Feb 16;12(4):847. doi: 10.3390/foods12040847 (PMC9957260; doi:10.3390/foods12040847)
Supplement: Supplementary file 1 [file foods-12-00847-s001.zip › foods-2183937-supplementary.pdf]

SUPLEMENTARY MATERIAL (Figure S1)

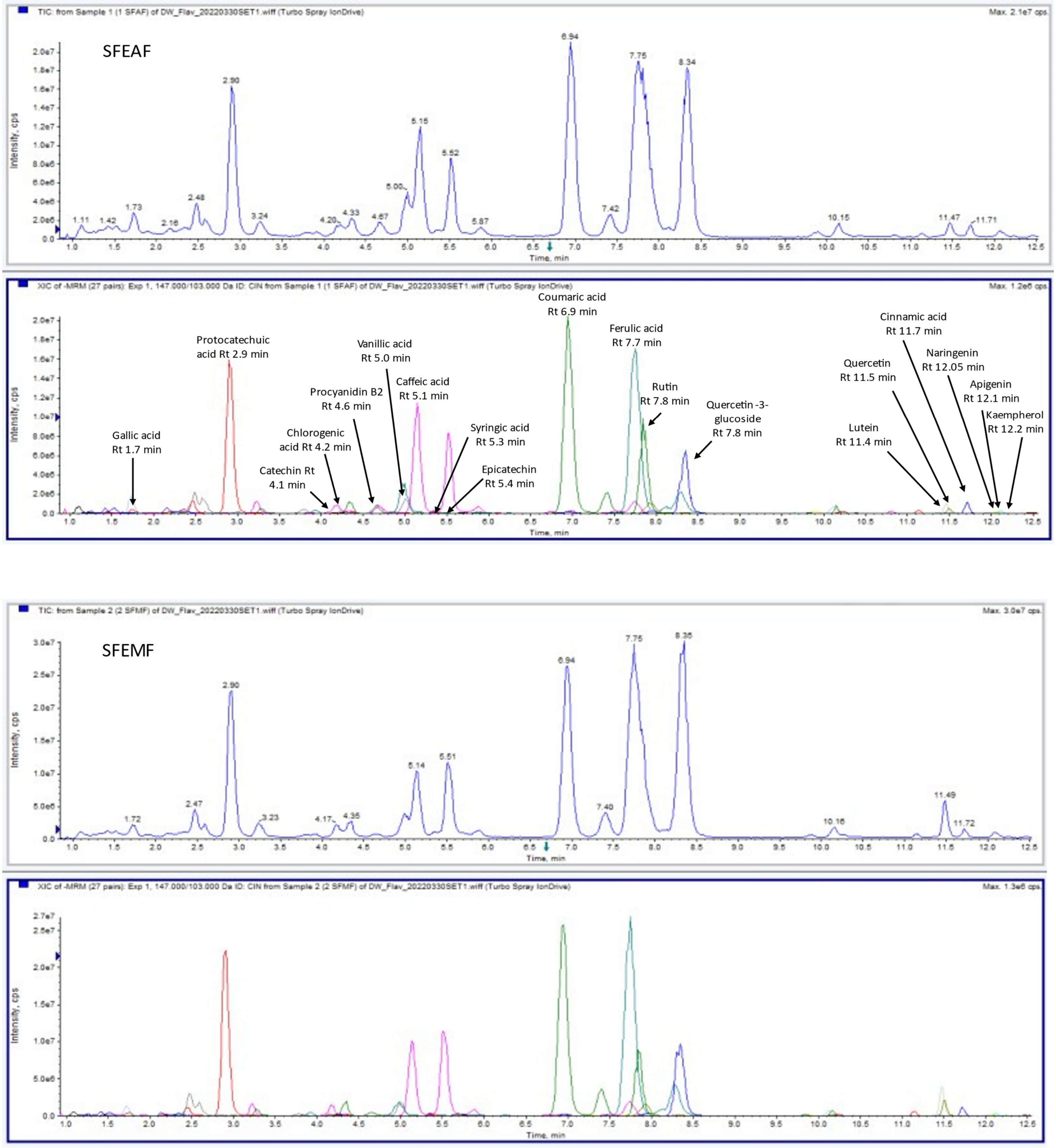

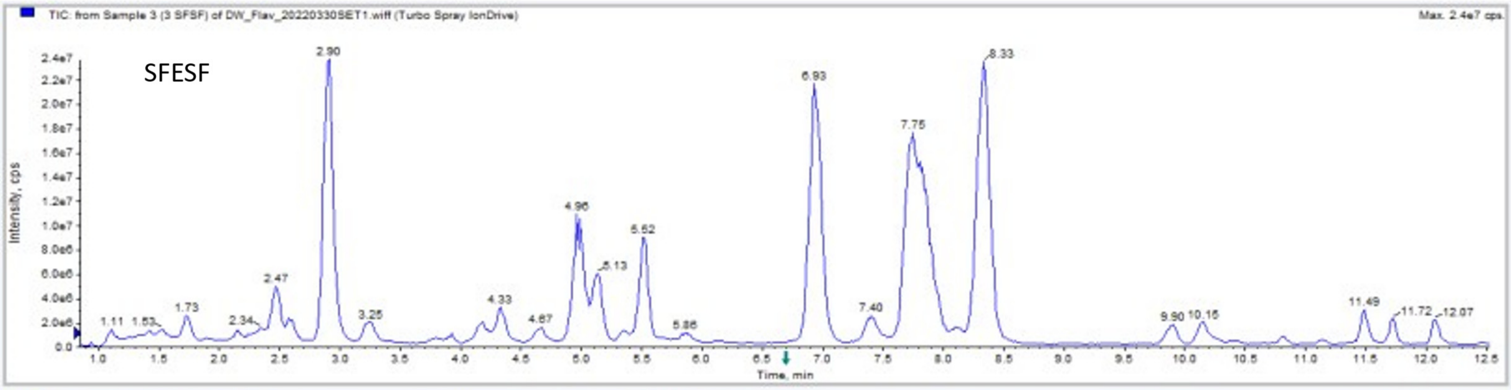
TIC: from Sample 3 (3 SFSF) of DW_Flav_20220330SET1.wiff (Turbo Spray IonDrive)
Max. 2.4e7 cps.
SFESF
Intensity, cps
Time, min

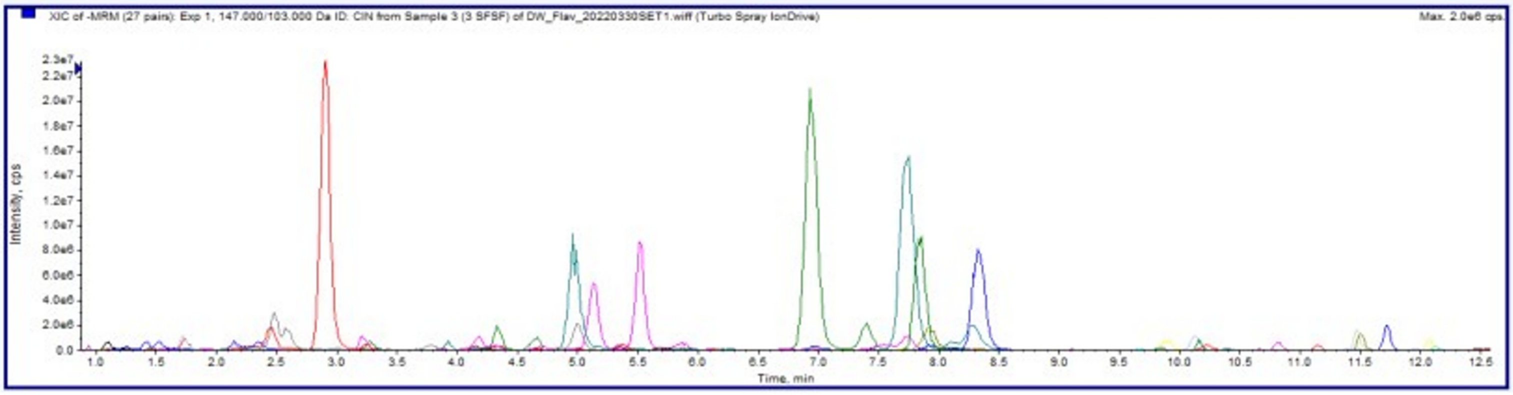
XIC of -MRM (27 pairs): Exp 1, 147.000/103.000 Da ID: CIN from Sample 3 (3 SFSF) of DW_Flav_20220330SET1.wiff (Turbo Spray IonDrive)
Max. 2.0e6 cps.
Intensity, cps
Time, min

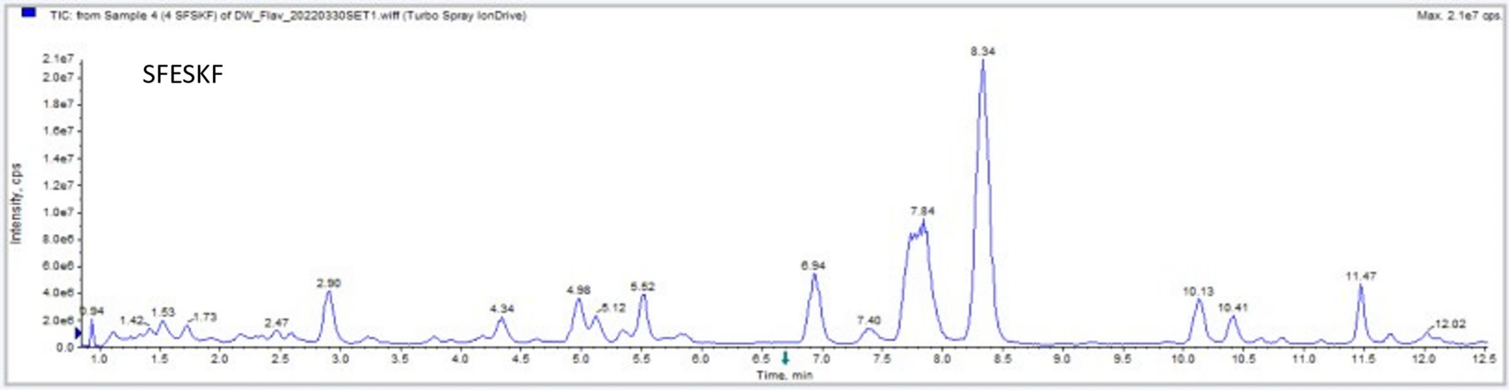
TIC: from Sample 4 (4 SFSKF) of DW_Flav_20220330SET1.wiff (Turbo Spray IonDrive)
Max. 2.1e7 cps.
SFESKF
Intensity, cps
Time, min

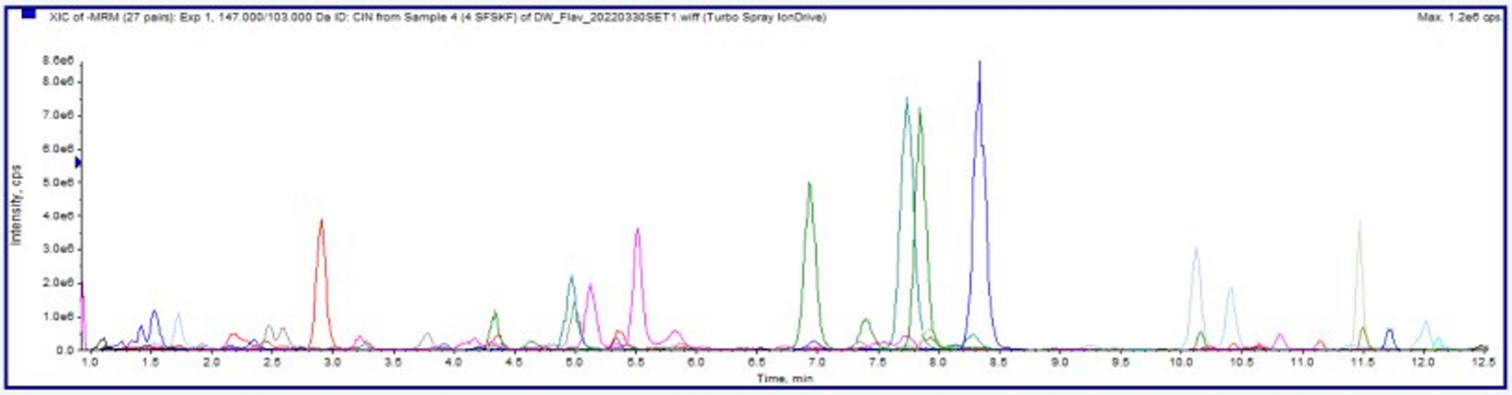
XIC of -MRM (27 pairs): Exp 1, 147.000/103.000 Da ID: CIN from Sample 4 (4 SFSKF) of DW_Flav_20220330SET1.wiff (Turbo Spray IonDrive)
Max. 1.2e6 cps.
Intensity, cps
Time, min

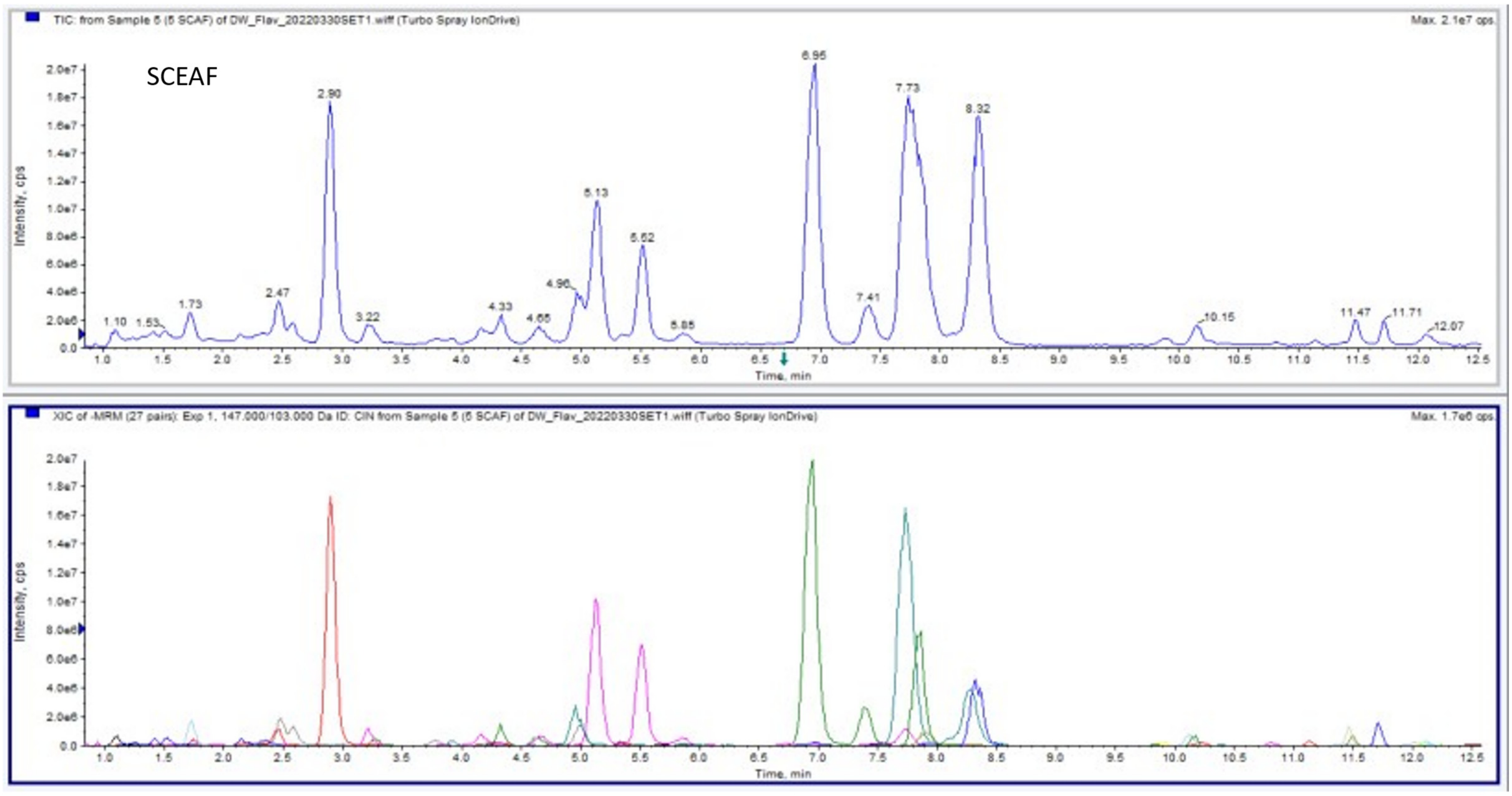
TIC: from Sample 5 (5 SCAF) of DW_Flav_20220330SET1.wiff (Turbo Spray IonDrive)
Max. 2.1e7 cps.
SCEAF
Intensity, cps
Time, min
XIC of -MRM (27 pairs): Exp 1, 147.000/103.000 Da ID: CIN from Sample 5 (5 SCAF) of DW_Flav_20220330SET1.wiff (Turbo Spray IonDrive)
Max. 1.7e6 cps.
Intensity, cps
Time, min

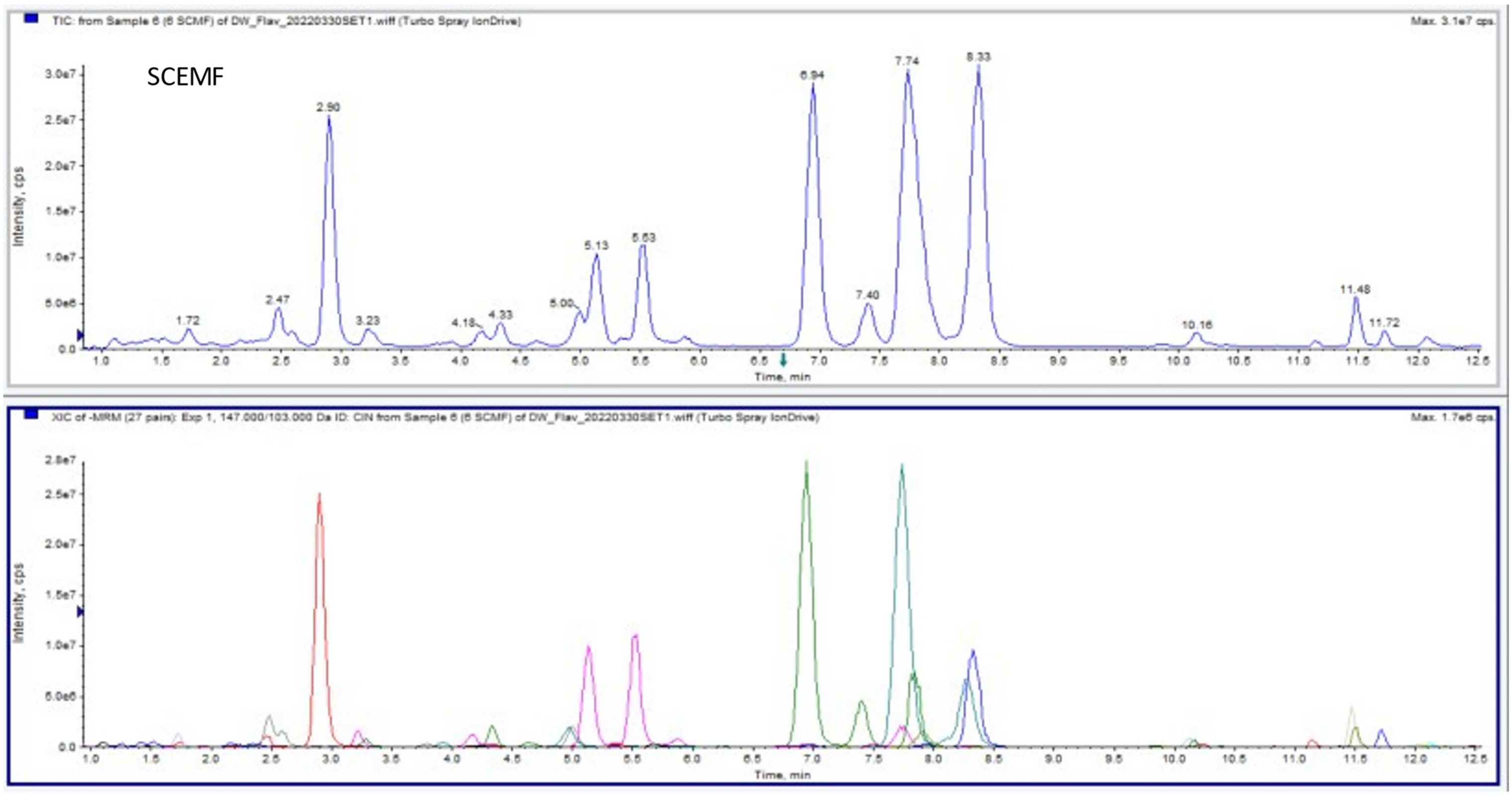
TIC: from Sample 6 (6 SCMF) of DW_Flav_20220330SET1.wiff (Turbo Spray IonDrive)
Max. 3.1e7 cps.
SCEMF
Intensity, cps
Time, min
XIC of -MRM (27 pairs): Exp 1, 147.000/103.000 Da ID: CIN from Sample 6 (6 SCMF) of DW_Flav_20220330SET1.wiff (Turbo Spray IonDrive)
Max. 1.7e6 cps.
Intensity, cps
Time, min

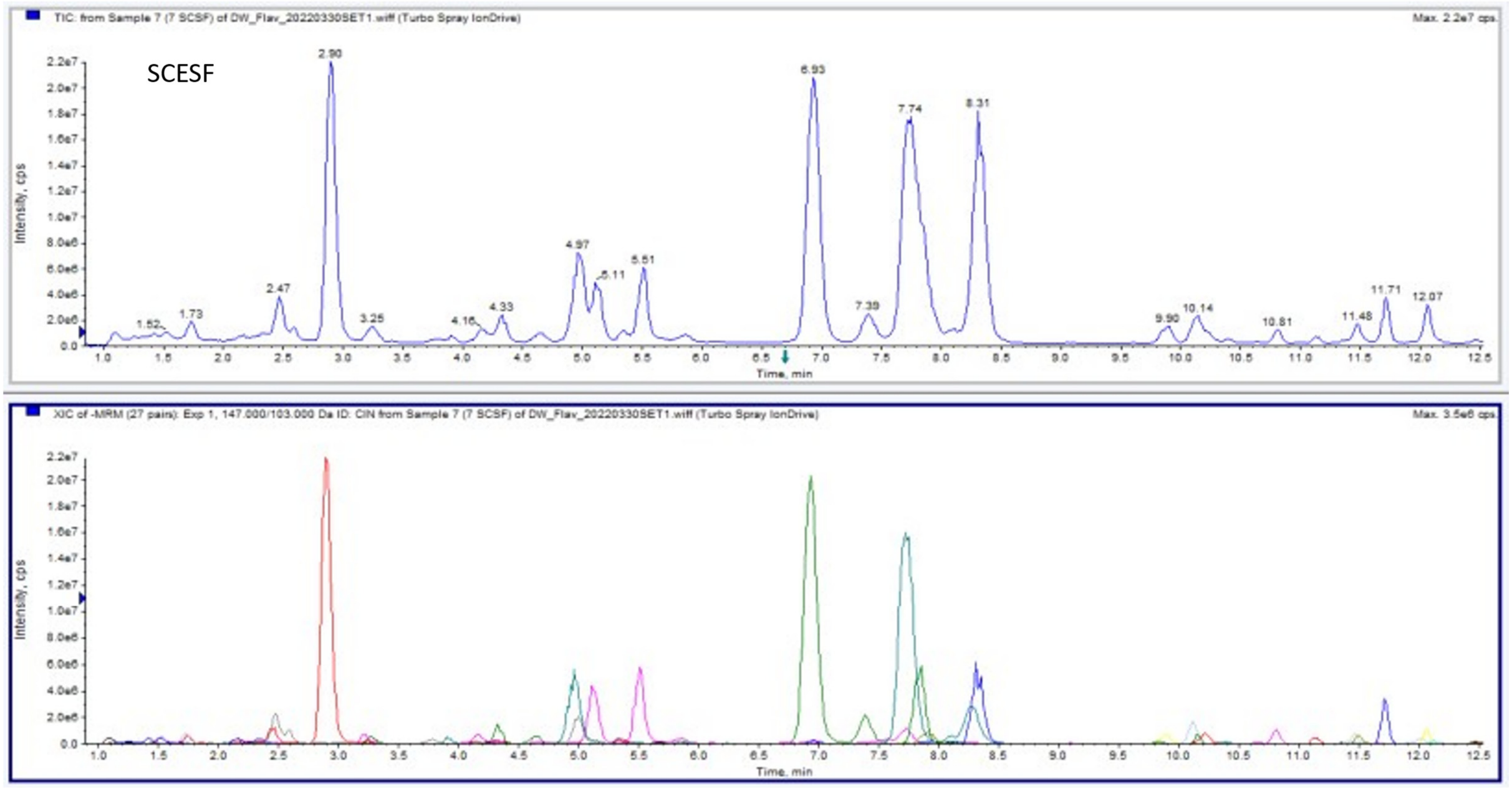
TIC: from Sample 7 (7 SCSF) of DW_Flav_20220330SET1.wiff (Turbo Spray IonDrive)
Max. 2.2e7 cps.
SCESF
XIC of -MRM (27 pairs): Exp 1, 147.000/103.000 Da ID: CIN from Sample 7 (7 SCSF) of DW_Flav_20220330SET1.wiff (Turbo Spray IonDrive)
Max. 3.5e6 cps.
Intensity, cps
Time, min

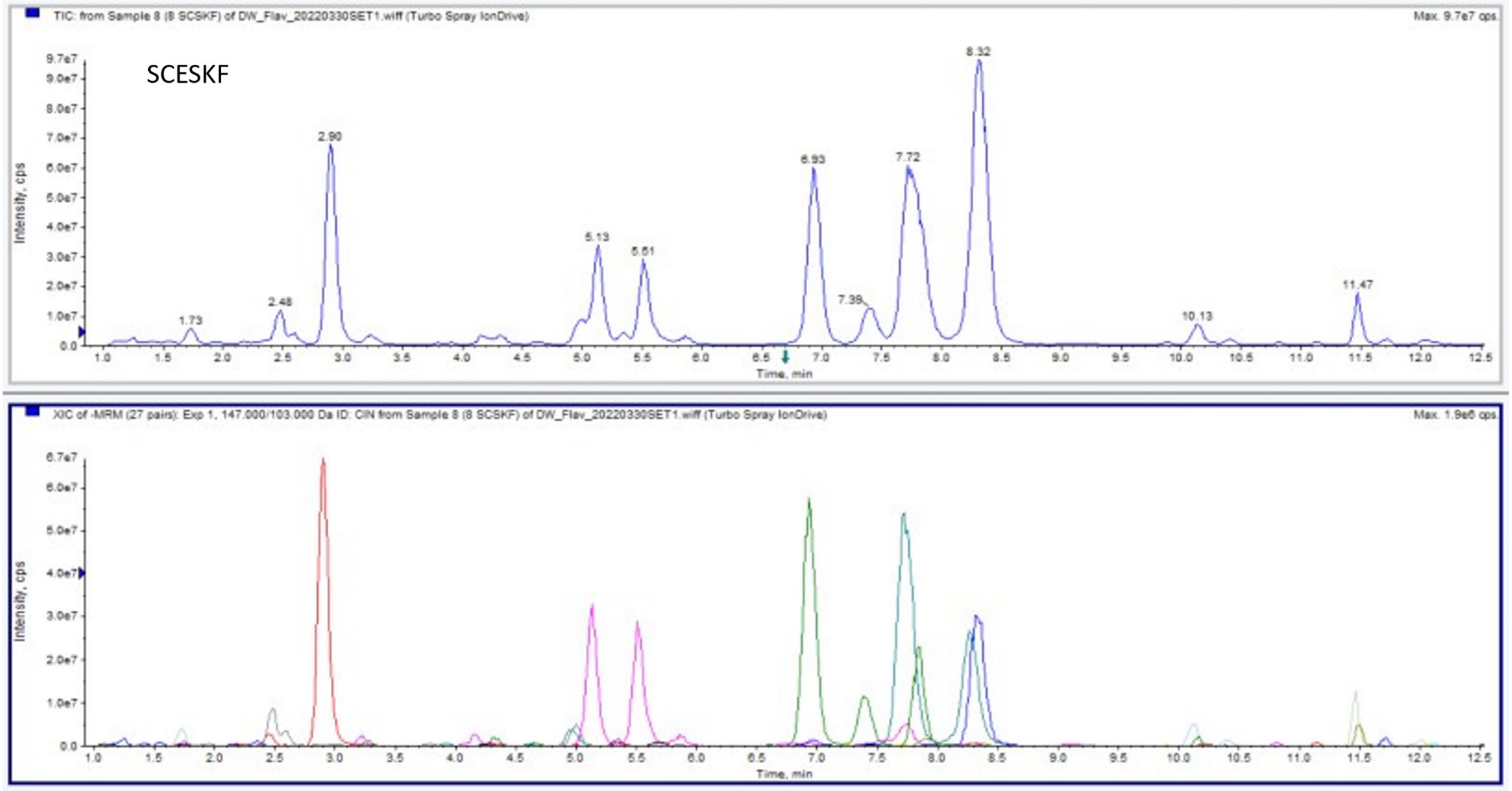
TIC: from Sample 8 (8 SCSKF) of DW_Flav_20220330SET1.wiff (Turbo Spray IonDrive)
Max. 9.7e7 cps.
SCESKF
XIC of -MRM (27 pairs): Exp 1, 147.000/103.000 Da ID: CIN from Sample 8 (8 SCSKF) of DW_Flav_20220330SET1.wiff (Turbo Spray IonDrive)
Max. 1.9e6 cps.
Intensity, cps
Time, min

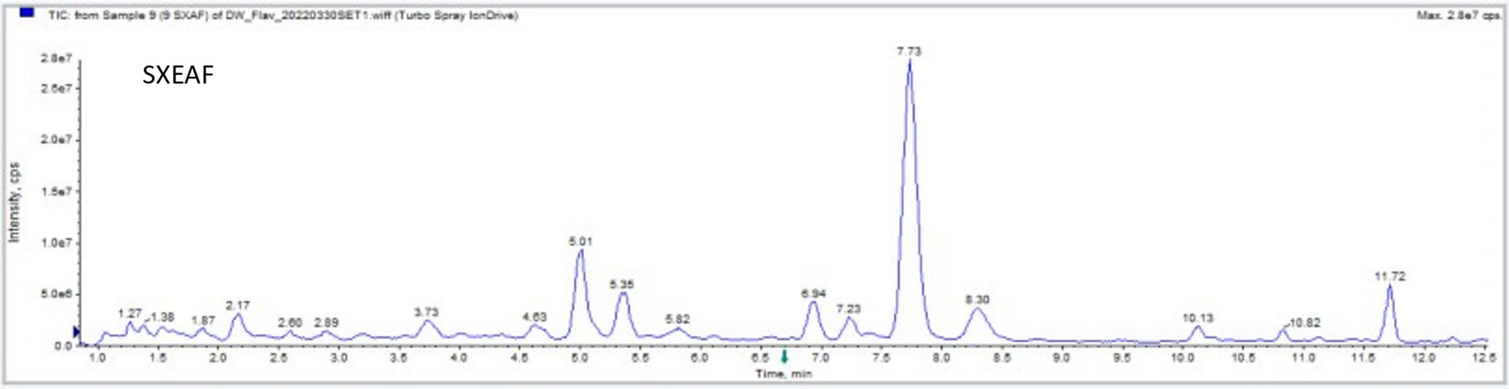
TIC: from Sample 9 (9 SXAF) of DW_Flav_20220330SET1.wiff (Turbo Spray IonDrive)
Max. 2.8e7 cps.
SXEAF
Intensity, cps
Time, min
1.27
1.38
1.87
2.17
2.60
2.89
3.73
4.63
5.01
5.35
5.82
6.94
7.23
7.73
8.30
10.13
10.82
11.72

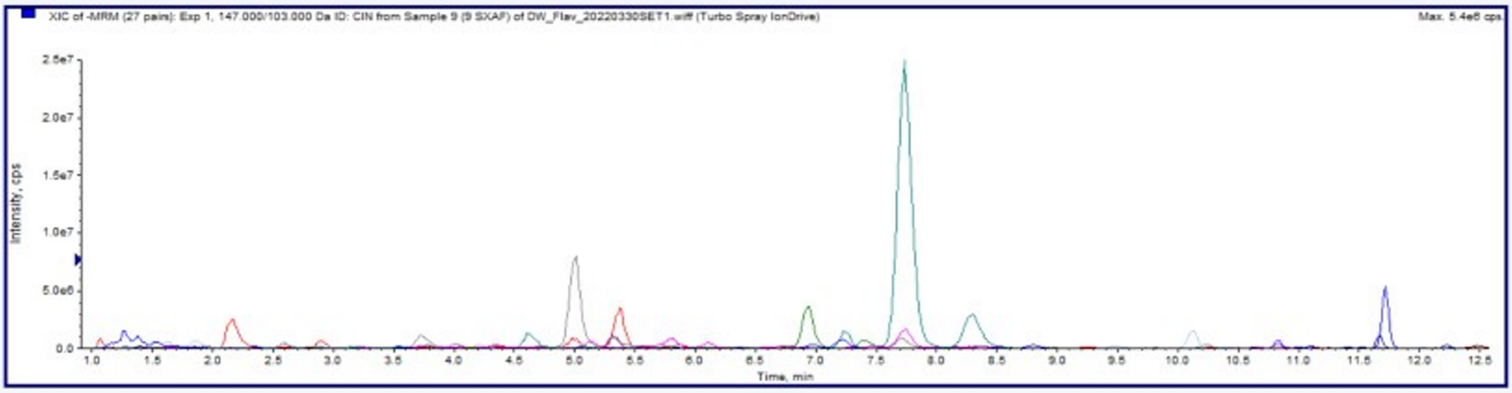
XIC of -MRM (27 pairs): Exp 1, 147.000/103.000 Da ID: CIN from Sample 9 (9 SXAF) of DW_Flav_20220330SET1.wiff (Turbo Spray IonDrive)
Max. 5.4e6 cps.
Intensity, cps
Time, min

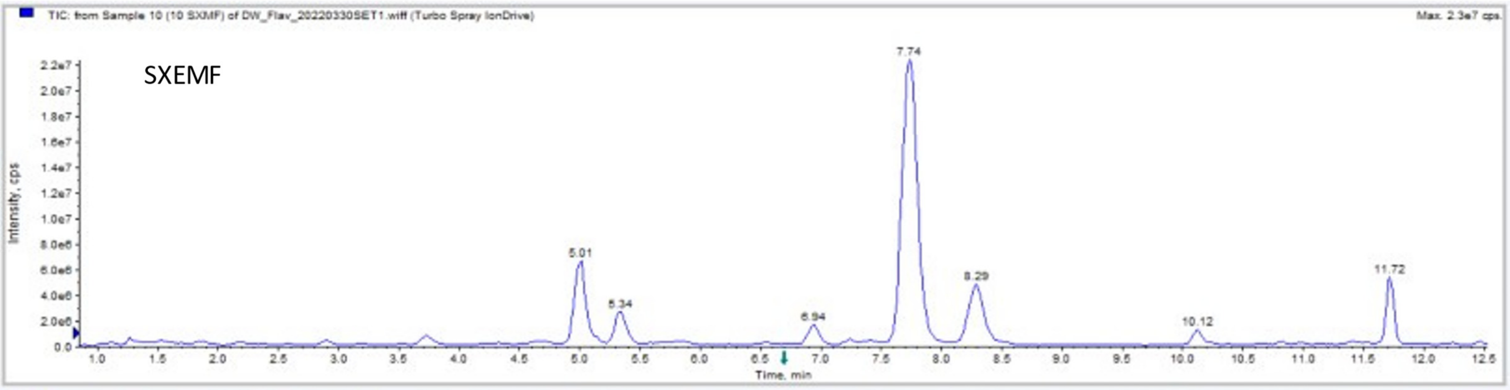
TIC: from Sample 10 (10 SXMF) of DW_Flav_20220330SET1.wiff (Turbo Spray IonDrive)
Max. 2.3e7 cps.
SXEMF
Intensity, cps
Time, min
5.01
5.34
6.94
7.74
8.29
10.12
11.72

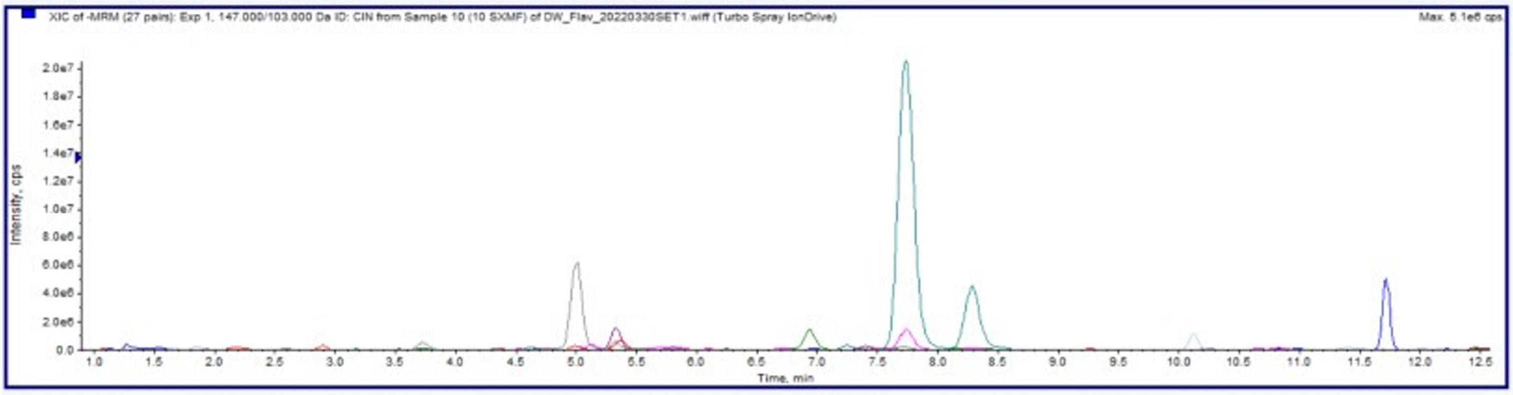
XIC of -MRM (27 pairs): Exp 1, 147.000/103.000 Da ID: CIN from Sample 10 (10 SXMF) of DW_Flav_20220330SET1.wiff (Turbo Spray IonDrive)
Max. 5.1e6 cps.
Intensity, cps
Time, min

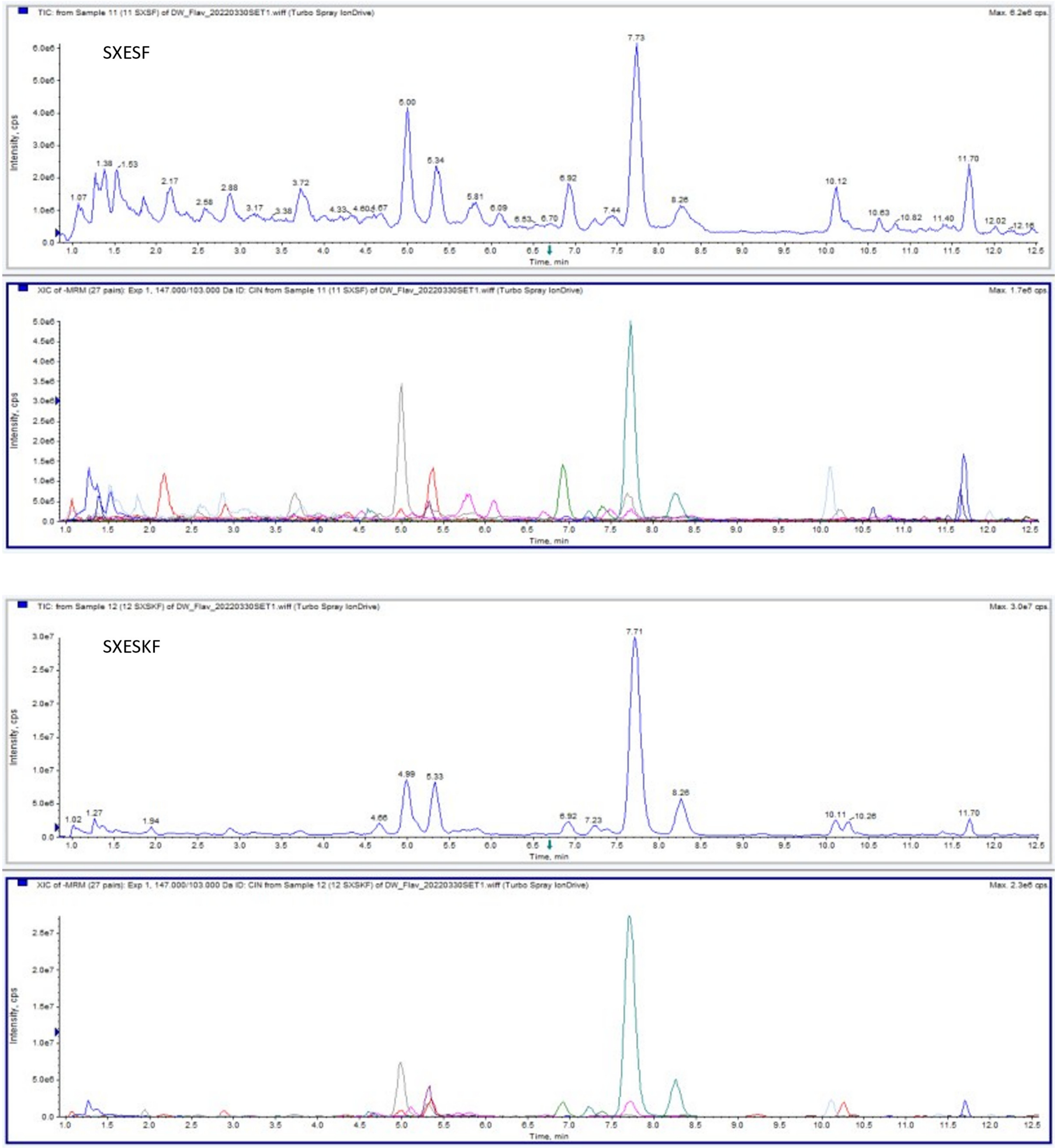

Figure S1: LC/MS chromatogram for phenolic acids and flavonoids in extracts from date flesh of four date fruit varieties (Ambara flesh (AF), Majdool flesh (MF), Sagai flesh (SF) and Sukari flesh (SKF)) using supercritical fluid (SFE), subcritical CO_2 (SCE) and Soxhlet (SXE) extraction methods.
